# Immunotherapy of Oncovirus-Induced Cancers: A Review on the Development and Efficacy of Targeted Vaccines

**DOI:** 10.3390/vaccines13090911

**Published:** 2025-08-27

**Authors:** Chi Sing Ng

**Affiliations:** Department of Pathology, Caritas Medical Center, Kowloon, Hong Kong; ngcspeter@gmail.com; Tel.: +(852)-3408-7756; Fax: +(852)-2745-1804

**Keywords:** oncogenic viruses, vaccines, prevention, therapy

## Abstract

Background: A number of viruses are oncogenic. These include the human papilloma virus (HPV), Epstein–Barr virus (EBV), Kaposi sarcoma human herpes virus 2/human herpes virus 8 (KSHHV/HHV8), hepatitis B virus, (HBV), hepatitis C virus (HCV), Merkel cell polyoma virus (McPyV), and the human T-cell leukemia virus type 1 (HTLV-1). These viruses cause malignancies ranging from carcinomas, sarcomas, lymphomas, to leukemias. This review aims to study the effects and efficacy of vaccines against these viruses and the cancers they cause in their prevention and treatment. Methods: The literature in the past 30 years was searched employing Scopus and Google Scholar using the keywords “oncogenic viruses, HPV, EBV, KSHHV, HHV8, Polyoma virus, HTLV-1, COVID-19, carcinoma, sarcoma, lymphoma, leukemia, anti-virus vaccines”. Results: Prophylactic vaccines against the HPV and HBV are highly effective in preventing and reducing the incidence of uterine cervical and hepatocellular carcinomas. Prophylactic vaccines against other oncogenic viruses have been less successful, though efficacious in some experimental animals. Therapeutic vaccines are still mostly under evaluation and development. Conclusions: Identification of oncogenic viruses has rendered anti-viral vaccines conspicuous tools for preventing and treating cancers they cause. Many endeavors for the development of such vaccines have been met with limited success, apart from the very effective anti-HPV and anti-HBV vaccines in universal vaccination programs. With the development of new vaccine technologies, it is hoped that effective vaccines against other oncogenic viruses will be developed in the future.

## 1. Introduction

Cancers attributed to infections account for 13–15% (about 2.2 million cases) of all human cancers, with viruses accounting for more than 60% of infection-attributed cancers in 2018 [1,2,3,4,5] (Table 1). Oncogenic viruses contribute to cancer development by acting as oncogenes or epigenetically through oncogene activation, tumor suppressor gene inactivation, dysfunctional transcription, interfering with cell signaling pathways, and triggering oncogenic-enabling events in the microenvironment, like inflammation [6,7,8,9]. Oncogenic viruses are among the rare known causes of cancer [6,10] and are often regarded as “low-hanging fruits” amenable to cancer prophylaxis, diagnosis, prognostication, and therapy [6,11,12]. Furthermore, most humans are infected with some of these viruses, such as the ubiquitous EBV [6,7]. Prophylaxis of the viral infections, especially latent infections, is an effective and economical means to cancer prevention and management [6,7,10,11,12]. Due to the prevalence of these viruses and their significant role in human oncogenesis, development and application of vaccines to prevent the infections and treat the virally caused cancers has attracted much interest and achieved variable success [11,12,13,14]. This review is geared to reviewing viral oncogenesis and the application of vaccinology in the prevention and treatment of the virally caused human cancers.

## 2. Vaccine Technologies and Platforms

The goal of vaccination is to induce an immunological response to insulting agents [15,16]. Under the scope of this review, only oncogenic viruses and the established related cancers are discussed. The vaccination strategies include prophylaxis, thus averting both lytic and latent infections, and therapeutic interventions for established cancers. The gist of vaccination is to introduce relevant insulting protein/antigen targets, including viral or cancer cell-derived proteins, into humans to elicit immune humoral and cellular responses. An effective vaccine technology and platform is successful in presenting these antigens/proteins to immune cells to elicit effective, robust immune cascades, production of specific neutralizing antibodies, and cellular immunity. The immune cells involved include antigen-presenting cells (APCs), dendritic cells (DCs), macrophages, B-cells, T-cells, and natural killer (NK) cells [15,16].

### 2.1. Anti-Viral Vaccines

#### 2.1.1. Protein Platform

##### Whole Inactivated (Killed) Vaccines

This involves the inactivation/killing of whole viruses by chemical or physical means or both [15,17]. The commonly used chemicals are formaldehyde, glutaraldehyde, ascorbic acid, hydrogen peroxide, beta-proiolactone, and ethylenimine derivatives. Physical means commonly employed are heat, ultraviolet light, and gamma irradiation [16,17]. These vaccines are safe, particularly in immunocompromised subjects, as the pathogenic viruses have been killed. They, however, produce weaker immune responses compared to live pathogens. Adjuvants are, therefore, usually required for enhancing the immunogenicity of these vaccines. Nevertheless, elicitation of immune responses to more viral protein targets in these whole inactivated vaccines compared to subunit vaccines may be produced. Vaccine production is, however, time-consuming, more expensive, and less amenable for a rapid response to fight emerging outbreak pathogens [15,17].

##### Live Attenuated Vaccines

Viruses are attenuated through serial passaging under suboptimal conditions or temperatures and using genetic engineering methods that increase replication fidelity or codon de-optimization. These methods select mutations that disable their viral pathogenic capacities [15,17,18,19,20]. Live attenuated vaccines have enhanced immunogenicity and elicit potent cellular and innate responses. Most do not require adjuvants, and a single vaccine dose can drive lifelong immunity. The disadvantages are their residual minor pathogenic potential, especially in immunocompromised subjects [15,17,21,22], and the labor-intensive and costly production [15,17,23].

##### Virus-like Particle (VLP) Vaccines

VLPs are macromolecular assemblies of viral structural components, meant to imitate the morphology and protein structure of the virus. VLPs are bio-manufactured after transfection of bacterial, yeast, insect, plant, or mammalian cells with one or more genetic constructs of the virus, resulting in self-assembly into incomplete viral protein particles [24]. Fine-tuning of VLP immunogenicity is possible through surface chemical modification and adding immunogenic peptides and adjuvants [25]. VLP vaccines are effectively targeted by B-cells, DCs, and APCs. Interaction within and increased avidity for innate immune cells increase VLP vaccine immunogenicity [26]. Manufacturing costs are, however, higher.

##### Synthetic Peptide Vaccines (SPVs)

SPVs contain immunodominant viral peptides that require extensive in vitro screening and modeling to discover. SVPs are synthesized using fragment condensation or solid-phase synthesis. These small peptides are mixed with or conjugated to carefully chosen adjuvants to avoid peptide denaturation and to enhance uptake by APCs. SPVs are safe and cause focused epitope-specific immune responses, though epitope restriction reduces response breadth and may result in escape variants of the virus [15,17].

#### 2.1.2. Nucleic Acid Platforms (NAPs)

NAPs use selected nucleic acid sequences to generate viral proteins and antigens that are not disease-causing, with production of potent host immune responses [15,17]. Modular designs in NAPs enable their application in outbreaks with rapid and large-scale manufacturing at lower costs. NAPs are useful against emerging and new pathogens, pandemic threats, and escape viral variants [15,17].

##### Bacterial and Viral Vectored Vaccines

These vaccines utilize non-pathogenic or attenuated pathogenic bacteria and viruses as carriers of nucleic acid sequences of the target pathogenic virus. Bacterial vectored vaccines are suitable for mucosal administration. Though there is a risk of infection by the bacterial vector in the immunocompromised, the risk is much mitigated by identification and deletion of bacterial virulence genes with genetic engineering. Viral vectors retain infectivity and express the encoded target antigen. Replication incompetent vectors do not cause productive infection and are safe and easy to manufacture. Examples of bacterial vectors are non-pathogenic Lactobacillus sp. and attenuated Yersinia pestis. Some viral vector examples are vesicular stomatitis virus, vaccinia, adenovirus, poxvirus, and Newcastle disease virus [15,17,27,28,29].

##### Synthetic DNA Vaccines (SDNAVs)

SDNAVs are large, polyanionic, and nuclease sensitive and require special methods of delivery to recipients, including the more commonly used electroporation and nanoparticle systems [15,30]. A SDNAV is delivered into muscle tissue, leading to transfection of recipient cells with DNA translocation to nuclei, mRNA transcription, and target protein translation. The SDNAV stimulates both humoral and cellular immunity, causing robust immunogenicity and sustained potent responses. The theoretical risk of viral DNA integration into the recipient cell genomic DNA has been shown to be speculative [31].

##### mRNA-Based Vaccines (mRNAVs)

The drawbacks are RNA short half-life and reduced protein translation, aggravated by the recipient’s inflammatory response, which are circumvented by loading the mRNA into liposomes [15,17,32]. Recent technological advances, such as the incorporation of modified nucleosides into in vitro transcribed mRNA, sequence engineering, codon optimization, and utilization of safe lipid nanoparticle delivery systems, further enhance the stability of mRNAVs [33,34]. There are three main categories of mRNAVs: conventional, self-amplifying (SA), and circular (c) RNA. Extended protein translation is possible in SA mRNA due to viral-derived molecular machines that enable intracellular mRNA sequence amplification [15,35], and in cRNAVs through the addition of interval ribosomal entry sites [15,36]. mRNAVs elicit potent cellular and humoral immunity, which is partly due to their delivery systems such as nanoparticles [32,37]. There is no risk of genomic DNA integration. They are easy to manufacture but require ultralow temperatures to store and transport.

### 2.2. Anti-Cancer Vaccines (ACVs)

ACVs are therapeutic vaccines developed against established cancers, including virus-induced cancers. As for anti-viral vaccines, the aim of ACVs is to elicit potent immunity, mostly cellular immunity, against cancer cell antigens that are exogenously or endogenously introduced. ACVs may also augment host immunity or induce changes in the tumor microenvironment (TME). The former requires cancer antigens for presentation to DC, helper and cytotoxic T-cells, and other cellular constituents of the TME. Another concern of ACVs is the possible development of intrinsic tumor resistance to the vaccine due to cancer cell genomic instability with alteration in expression and processing of tumor antigens [16,38,39]. In view of this, antigen-agnostic in situ vaccines (ISVs) that modify and augment immune robustness in the TME and provide directly tumor-associated antigens by inducing in situ cancer cell death were developed [16,40]. ISVs, however, may also be compromised by tumor resistance through mechanisms of interfered T-cell functions, development of inhibitory cytokines/regulatory cells, and remodeled extracellular matrix [16,41,42].

#### 2.2.1. ACV Technologies and Platforms

##### Tumor Antigens

The quality of tumor antigens is at the center stage of ACVs for robust effectiveness and efficacy. There are two commonly used categories of antigens.

(a) Neoantigens

Neoantigens are mutated tumor antigens produced through non-synonymous somatic and frameshift mutations and in coding regions, human endogenous retroviruses, post-translational protein phosphorylation, citrullination, and glycosylation [16,43,44,45,46]. This is usually applicable to tumors with high tumor mutation burden (TMB), which presumably provide large numbers of tumor-rejection neoantigens for vaccine targeting [16,47].

(b) Shared antigens

These ACVs utilize several shared tumor-associated antigens, usually applicable to tumors with low TMB [16,48,49].

##### ACV Platforms

These include direct antigen administration using neoantigens, RNA, and synthetic long peptides. They could be administered intramuscularly, intravenously, by antigen delivery through loaded DCs or non-cellular particles, nanoparticles, lipoplexes, amphophilic systems, liposomes, or polyethy-eneimine silica microrods [16,50,51,52].

##### In Situ Vaccines (ISVs)

ISVs are antigen-agnostic and not targeted at any individual tumor antigen. They aim to enhance endogenous anti-tumor responses by affecting the TME or causing anti-tumor effects at distal sites in addition to the local site of treatment by abscopal effect [16,40]. The TME cellular targets include APCs, DCs, and T-cells, resulting in proliferation, activation, and maturation of the cells, leading to a robust anti-cancer cell immune response. Oncolytic viruses (such as herpes simplex virus, parvovirus, and adenovirus) may be employed, which are effective due to activation of immune cells and induction of tumor cell death, thus releasing tumor-associated antigens. Further, oncolytic viruses enriched for the toll-like receptor agonist CpG (cytosine–phosphate–guanine) motifs strongly activate APC functions [16,40,53,54]. Oncolytic viruses administered to the TME in combination with agents that suppress the induction of immunosuppressive cells (regulatory T-cells) also contribute to enhanced anti-tumor immunity. The agents used in combination with oncolytic viruses may include cyclophosphamide, temozolomide, and immune checkpoint blocking antibodies [40,55,56,57,58].

##### Combined Vaccination

This strategy may be employed in advanced cancers as part of a multiphasic approach (surgery, chemotherapy, radiotherapy, and vaccination) [16].

### 2.3. Adjuvants

Adjuvants are substances that enhance vaccine immunogenicity. While inactivated vaccines contain adjuvants introduced during vaccine production, most modern vaccines contain small components of the target proteins/antigens and may require additional adjuvants. Adjuvants range from aluminum salts, emulsions, liposomes, nanoparticles, to VLPs. Adjuvants target innate immune cells and activate pattern recognition signaling pathways [59,60,61]. mRNA vaccines further possess intrinsic adjuvant activity due to the nucleoside-unmodified RNA (in particular in double-stranded RNA), which triggers innate immune signaling [55]. The delivery systems (such as lipid nanoparticles) used in modern vaccines are also contributory adjuvants [59,60,61].

## 3. The Oncogenic Viruses, Attributed Tumors and Vaccines

Viruses may cause cancer by acting as oncogenes, activating host oncogenes, creating host genomic instability, inhibiting host tumor suppressor genes and proteins, interfering with cellular signaling pathways, increasing cell proliferation, resisting cell apoptosis, affecting DNA repair, contributing to tumor evasion through genetic or epigenetic mechanisms, and causing chronic inflammation [2,3,6,7,8,9]. Oncogenic viruses are responsible for more than 60% of infection-attributed cancers worldwide in 2018 [1,2,3]. The best-known seven oncogenic viruses are described in the following sections. They may act as solos, duets, or trios in the virus orchestra, with the coinfections contributing to higher cancer incidence or more advanced disease. The human immunodeficiency virus (HIV) is, per se, not oncogenic but plays an important role in cancer development when playing in the orchestra [1,2,3,6,7]. The SARS-CoV-2 virus, postulated to be possibly oncogenic, is also discussed below.

### 3.1. Human Papilloma Virus (HPV)

#### 3.1.1. Virology and Oncogenesis

The HPV is a non-enveloped DNA virus with a double-stranded genome containing about 8000 base pairs. The genome is enclosed in an icosahedral capsid made up of the structural proteins L1 and L2, which are encoded by the late region of the HPV genome. Non-structural proteins E1, E2, E3, E4, E5, E6, and E7 are encoded by the early region, and E6 and E7 are most essential for oncogenesis. The HPV belongs to the family Papillomaviridae, in which more than 400 HPV genotypes have been identified and categorized into the five genera: Alpha, Beta, Gamma, Mu, and Nu. The Alpha HPVs contribute most to oncogenesis. HPV genotypes are classified according to their oncogenic potential into high-risk (hrHPV) or low-risk (lrHPV). The hrHPV are 16, 18, 31, 33, 35, 39, 45, 51, 52, 56, 58, 59, 66, and 68. HPV16 and 18 are responsible for more than 70% of global cervical cancer cases [4,5,62,63].

The prototype of HPV oncogenesis is uterine cervical cancer. Cervical cancer is the fourth leading cause of cancer deaths in women in 2020 [62,63]. HPV infection is acquired through the basement membrane of the affected epithelium and infects the basal cells, where it remains in episomal form. It replicates in the upper epithelial layers, exploiting the host tissue renewal mechanism. Most HPV infections resolve spontaneously. Persistent infection, however, results in disease and may lead to cervical intraepithelial neoplasia (CIN) or invasive cancer, especially by HPV16 and 18. In most cases, there is integration of viral DNA into the host genome, causing genomic instability, gene alteration, loss of tumor suppressor gene function, chromatin reorganization, chromosomal rearrangement, and epigenetic dysregulation. HPV integration usually occurs at hotspots (site-specific susceptibility) in the host genome. Examples are *FH1T*, *KLF5*, *LINC00392*, and *MACROD2. MACROD2* dysfunction can cause dysregulation of *PAR1*, which is involved in cell differentiation, proliferation, and tumor transformation. HPV integration is also dependent on the host and other viral cofactors. These may be the cervicovaginal microenvironment and microbiota, smoking, estrogen level, mental stress, and coinfection with HIV.

HPV integration disrupts the E2 open-reading frame, causing loss of E1/E2 interaction for initiating viral replication, dysfunctional E2 regulation of the viral early promoter, and overexpression of oncoproteins E6 and E7. The integration percentage increases with disease progression from CIN to cervical cancer. Overexpression of E6 and E7 further promotes HPV integration in the journey to cancer development [1,2,3,4,5,62]. For the latter event, genomic alterations involving the PI3K/MAPK and TGF-beta signaling pathways and mutations in *ERBB3*(*HER3*), *CASP8*, *HLA-A*, *SHKBP1*, and *TGFBR2* have been reported [63,64]. Despite HPV integration being the most important oncogenic event in cervical cancer, there are oncogenic mechanisms other than HPV DNA integration. The episomal E2, E4, and E5 oncogenic pathway [62,65], AP1/Wnt/beta-catenin pathway [62,66], PI3K/AKT/mTOR [62,66], and E2 methylation [62,67,68] are non-HPV integration mechanisms that have been reported in cervical cancer development. In one study, about 15% of cervical cancer specimens contained only the episomal form of HPV DNA, in which constitutively active *PIK3A* mutation, Myc overexpression, and RAS-MEK pathway activation are responsible for progression to tumorigenesis in vitro [69]. The advent of single-cell RNA sequencing (scRNA-seq) in recent years revealed novel cancer cell layers with dysregulated cell differentiation, extracellular matrix structure, and cell cycle dynamics. This sheds further light on the complex intercellular communications between HPV-positive cervical cancer cells and immune cells in the tumor microenvironment involving plasmacytoid dendritic cells, CD4+Th17/reg cells, and NK cells [70].

#### 3.1.2. HPV-Associated Cancers

About 91% of HPV-associated cancers in women are cervical cancers, which is the most common HPV-associated cancer subsequent to persistent infection in 5–15% of affected women [62,63]. Cofactors are often involved, including coinfection with HIV, Epstein–Barr virus (EBV), sexually transmitted diseases (Herpes Simplex, Chlamydia, Gonococcus), smoking, immunosuppression, young age at first pregnancy, hormone contraception, and multiparity [60,61,62,63]. The most frequently responsible hrHPVs are HPV16 and 18. Though approximately 90% of cervical cancers occur in low- and middle-income countries [61,63], the introduction of prophylactic HPV vaccination may potentially reshape its epidemiologic landscape.

**Figure 1:**
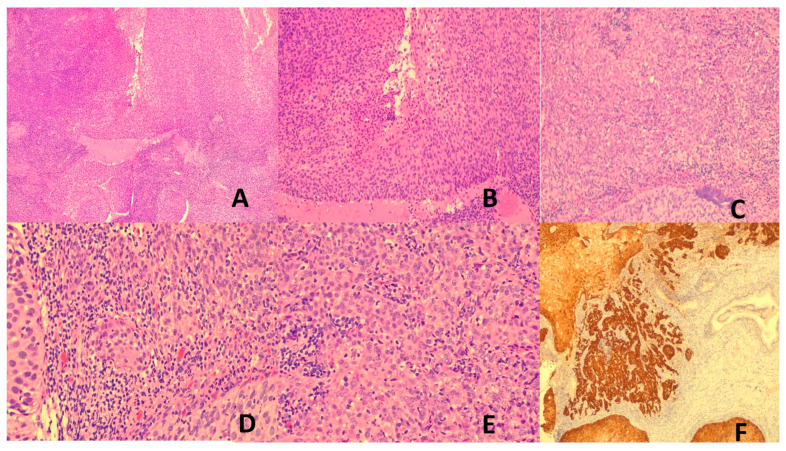
CIN3 and NKSCC of the cervix. F/40, vaginal bleeding. (**A**) CIN3 with glandular involvement, H&E ×100. (**B**) CIN3, H&E ×400. (**C**) NKSCC with co-existing CIN3 (lower right), H&E ×100. (**D**) SCC with CINs (left), H&E ×200. (**E**) SCC with inflammation, H&E ×400. (**F**) Positive p16 immunostaining, strong in carcinoma cells and moderate in CIN3 cells ×100.

HPV-associated cervical cancers include adenocarcinoma and squamous cell carcinoma (SCC). SCC is classified histologically as non-keratinizing, keratinizing, basaloid, condylomatous, papillary, or rarely lymphoepithelioma-like [71] (Figure 1). The prognosis of HPV-associated cervical cancer is better than that of the HPV-independent counterpart [71,72]. HPV-associated cancers also affect other anatomical sites. These include SCC of the anus, 15–48% of vulval carcinoma, 78% of vaginal carcinoma, 53% of penile carcinoma, 13–60% of oropharyngeal carcinoma, and <5% of laryngeal carcinoma [1,3,6], mostly caused by HPV16 [1]. Similar to cervical cancers, HPV-associated cancers of the head and neck region fare better than their HPV-independent counterparts [73].

#### 3.1.3. Vaccines

These include prophylactic vaccines for disease prevention and therapeutic vaccines to treat established cancers.

##### Prophylactic Vaccines (PVs)

PVs against HPV were first licensed in 2006. Currently, there are six licensed recombinant DNA PVs, manufactured based on purified L1 structural protein. The proteins produced from HPV type-specific empty shells or VLPs are potent immunogens bearing no pathogenic or infectious potential. They are fortified with adjuvants, and all are against HPV16 and 18. The vaccines are bivalent (HPV16 and 18), quadrivalent (HPV6,11,16,18), and nonavalent (HPV6,11,16,18,31,33,45,52,58) [1,74,75]. (Table 2). To date, 125 countries (64%) have introduced the HPV vaccine in their universal immunization program for girls, and 47 countries (24%) for boys [1]. PVs are aimed to be administered before sexual exposure and are indicated for females aged 9 or older. PVs against HPV have been found to substantially reduce the incidence of CIN3 and cervical cancer [1,12,74,75]. They are effective in HIV-positive subjects [1].

##### Therapeutic Vaccines (TVs)

TVs are based on mechanisms discussed under Section 2.2. Platforms employing long, naked DNA, RNA, peptides, proteins, viral and other microbial vectors, and antigen-presenting cells have been efficacious in patients with CIN. Evidence of efficacy is, however, limited in patients with HPV-attributed cancers [76,77]. Oncolytic viruses have been shown in preclinical settings to be an effective immunotherapy for HPV-associated cancers, stimulating anti-tumor immunity and cancer cell eradication [62,76]. However, no licensed TV against HPV+ cancers is available to date.

#### 3.1.4. Viral Coinfections

While HPV is the primary causative oncovirus and can cause cancer as a soloist on its own, coinfections with HIV and EBV, which aggravate the incidence and severity of the cancer caused, may act as duets or trios.

##### HPV and HIV

The incidence of HPV-associated anal, cervical, and oropharyngeal cancer is 80, 22, and 6 times higher in HIV-infected compared to HIV-uninfected subjects [78,79,80,81]. The incidence of oral, head and neck, liver, lung, testicular, and kidney cancers is also increased in HIV-infected individuals despite antiretroviral therapy (ART), probably related to potentially oncogenic proteins [82]. While HIV may increase the risk of cancer development by attenuating local and systemic immunity, it has been observed that HIV may induce epithelial–mesenchymal transition (EMT), which is a process of losing the epithelial characteristics of apical polarity, cell tight junctions, epithelial immunophenotype (such as E-cadherin), and cell adhesion. This is followed by expression of mesenchymal (such as vimentin) and stemness phenotypes (such as CD133 and CD44), dedifferentiation, cancer development, and invasiveness [81]. EMT is regulated by beta-transforming growth factor [83] and the MAPK [84] signaling pathways. Inhibition of these pathways may prevent HIV-associated acceleration of malignancy. It has been demonstrated that prolonged exposure of HPV-16 immortalized and HPV-uninfected epithelial cells to cell-free HIV-1 virions or the HIV-1 viral proteins gp120 and tat (trans-activator of transcription) results in promotion of EMT and cancer invasiveness [81]. Though HIV plays a role in HPV-associated cancers, the incidence of these cancers has not been reduced by ART in HIV-infected populations [80]. This may be due to the limited penetration of ART drugs into solid tissues [85]. Effective anti-HIV vaccines, however, have regrettably been unavailable [86].

##### HPV and EBV

Coinfection with EBV has been documented in HPV-associated CIN and cervical cancers [87,88,89,90,91]. While EBV has been considered a cofactor [89], it is also etiologically related to HPV-associated cervical cancers [90]. Coinfection with EBV increases HPV16/18 integration into the host genome [88,89,90], thus promoting cancer development [1,3,62]. EBV oncoproteins (such as Epstein–Barr nuclear antigen 1, EBNA1) promote EMT, angiogenesis, and activation of the signaling pathways NF-KB (nuclear factor kappa-light-chain-enhancer of activated B cells), MAPK (mitogen-activated protein kinase), and JAK/STAT (Janus kinase/signal transducers and activators of transcription). Other EBV latency products may also play a role. LMP1 (latency membrane protein 1) suppresses host immunity, and BARF1 (BAMHI-A rightward frame 1) promotes cell proliferation [3,90,91]. However, since EBV is a ubiquitous infection, it may be difficult to assess its involvement in promoting HPV carcinogenesis.

##### HPV, HIV, and EBV

Coinfections of the three viruses are observed in HPV-associated cancers [88,92,93]. They may play roles as primary factors or cofactors in oncogenesis.

### 3.2. Epstein–Barr Virus (EBV)

#### 3.2.1. Virology and Oncogenesis

EBV is a ubiquitous gamma herpes enveloped double-stranded DNA virus also known as human herpes virus 4 (HHV4). It infects over 90% of the world’s human population and spreads by oral secretions. Most infections occur during early childhood and are asymptomatic. Development of infectious mononucleosis is rare, though more frequent in well-developed countries [7,13,94]. After cell entry, EBV DNA enters the host cell nucleus and forms a stable episome for long-term survival and latent infection [7,94]. The latent infection is implicated in cancer development, and it is estimated that EBV-related malignancies are responsible for 0.2–0.3 million new cancer cases and 0.1–0.2 million cancer deaths in 2020 [94,95]. EBV is tropic for B lymphocytes and epithelial cells. Viral entry is through interaction with CD21 on B lymphocytes and integrin on epithelial cells [7,96]. EBV also infects NK and T-cells, probably mediated by CD21 acquired from infected B-cells by synaptic transfer [7].

There are three patterns of latency with differential expression of six nuclear proteins (EBNA1, 2, 3A, 3B, 3C, and leader protein EBNA-LP), three latent membrane proteins (LMP1, 2A, and 2B), BART mRNA, and EBER 1 and 2 (EBV small non-coding non-adenylated RNA). Restricted EBV products expression is present in latency 1 (EBNA1, EBERs, and BARTs) and latency 2 (LMP1, LMP2, EBNA1, EBERs, and BARTs). Latency 1 is observed in Burkitt’s lymphoma. Latency 2 expression is present in nasopharyngeal carcinoma (NPC), gastric cancer (GC), Hodgkin lymphoma (HL), and NK/T-cell lymphoma (NK/TL). Latency 3 expresses the full repertoire of EBV products and is observed in lymphoproliferative diseases associated with immunodeficiency.

Among the latency proteins, LMP1 is the major transforming protein mimicking constitutive CD40 receptor signaling, causing cell proliferation. Other EBV proteins interact with signaling pathways, cause *c-myc* activation and dysregulation, induce oxidative stress, suppress *MHC-II* gene expression, enhance PD-L1 expression, recruit SWI/SNF complex, and synergize with EZH2. This leads to cell proliferation, increased cell survival, decreased apoptosis, immune evasion, TME changes, cell transformation, and cancer development [7,8,94,95,97,98,99,100]. The six major signaling pathways involved in EBV oncogenesis are NF-KB, PI3K (phosphoinositide-3-kinase), JAK/STAT, MAPK, TGF-beta (transforming growth factor-beta), and Wnt/beta-catenin pathways [3,7,8,9,99,100,101].

Apart from latency products, EBV lytic products also contribute to oncogenesis [60,94,96,99,100,101]. There is a decrease in oncogenesis in cell cultures and experimental animals where the genes *BZL1*, *BRLF1*, and *BALF5* encoding early lytic proteins are knocked out. The early lytic proteins BZLF1, BRLF1, and BLLF3 increase expression of cytokines IL6, IL8, IL10, IL13, and IL1B, thus leading to immune modulation and evasion. BHRF1 and BALF1 are bcl2 homologs (vbcl2), and overexpression inhibits apoptosis. Early lytic proteins may also contribute to oncogenesis and invasiveness by causing genomic instability and angiogenesis. The lytic phase microRNAs (*BART* and *BHRF1-2*) contribute by inhibiting tumor suppressors (PTEN and PRDM1) and the ILR-1 receptor [3,97,99,102,103,104]. In recent years, scRNA-seq has demonstrated immunoglobulin (Ig) repertoire bias in EBV infection of B-cells. EBV subverts the processes that drive and regulate *Ig* rearrangement and mutation, physiologic selection of germinal center (GC) cells, and rescues Ig-deficient GC cells for survival [105].

#### 3.2.2. EBV-Associated Cancers

##### Nasopharyngeal Carcinoma (NPC)

**Figure 2:**
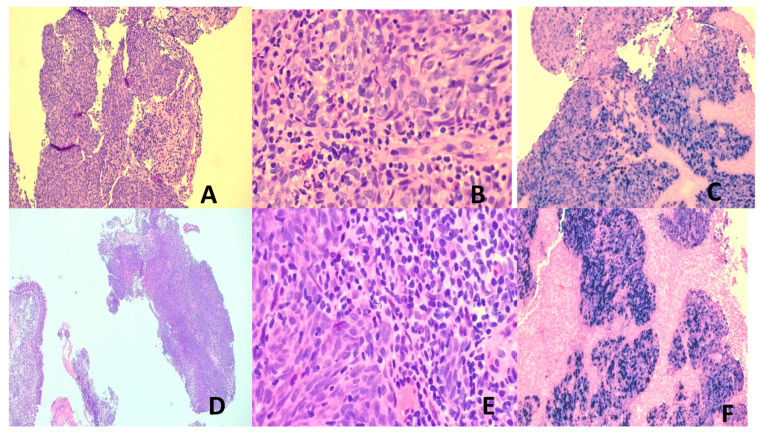
(**A**–**C**) NPC, M/40, post-nasal bleeding. (**A**) Syncytial epithelioid and spindle tumor cells, H&E ×100. (**B**) Syncytial sheets of plump spindle tumor cells, H&E ×400. (**C**) EBER-positive tumor cells. (**D**–**F**) LEC of lung, M/56, right lung nodule. (**D**) Spindle tumor cells, H&E ×100. (**E**) Sheets of plump spindle and epithelioid tumor cells, H&E ×400. (**F**) EBER-positive tumor cells ×100.

NPC is a rare cancer worldwide but shows high prevalence in southern China, south-east Asia, among Inuit populations in Alaska and Canada, and some populations in northern Africa (Algeria, Tunisia) [106,107]. It more commonly affects the NP lateral wall (especially the fossa of Rosenmuller) and may present with symptoms of an NP mass (blood-stained post-nasal drip, epistaxis, nasal obstruction), aural symptoms, diploplia, facial numbness, headache, or regional lymph node enlargement. There are three histological types: non-keratinizing SCC (NKSCC), keratinizing SCC (KSCC), and basaloid SCC. The NKSCC may show a rich intratumoral lymphoplasmacytic infiltrate, qualifying the designation “lymphoepithelioma-like carcinoma (LELC)”. NKSCC is characterized by invasive syncytia of large epithelioid or spindle tumor cells with indistinct cell membranes forming whorls, fascicles, or a reticular pattern [106] (Figure 2A–C). EBV is practically always associated with NKSCC with demonstrable ERBER. Positive EBV serology and elevated plasma EBV DNA are demonstrated in most NKSCC and are useful as an independent prognostic factor, marker of therapeutic response, tumor surveillance, relapse, and metastasis prediction [106,108]. Etiologically, the ethnicity prediction of NPC indicates genetic susceptibility with strong linkage of risk to MHC Class I variants (HLA-A, HLA-B, HLA-C) in Chinese patients [109], which may be related to dysfunctional immunosurveillance of EBV. Apart from the oncogenic mechanisms discussed before, interaction with host factors may also be important. Recognized host genetic factors are single-nucleotide polymorphisms in *MDS-EV11*, *TNFRSF19*, *CDKN2A*, *CDKN2B*, *TERTICLPTM1L*, and inactivated tumor suppressors *CDKN2A* and *TGFBR2* [104,110,111].

##### Gastric Cancer (GC)

EBV-associated GC accounts for <10% of GC worldwide, depending on the region and detection methodology [112,113,114,115,116]. This is designated gastric adenocarcinoma with lymphoid stroma in the WHO Classification [112], and is also referred to as LELC and medullary carcinoma [112,116]. It forms sheets, trabeculae, ill-defined tubules, or syncytia of large tumors with prominent lymphoid stroma. Other EBV-associated GC histologic subtypes have been reported, which differ in genetic alterations and PD-L1 expression [115].

EBV-positive GC is one of the molecular GC subtypes proposed by The Cancer Genome Atlas (TCGA) Research Network. It displays *PIK3CA*, *ARID1A* mutations, genome-wide hypermethylation, and amplification of the *CD274* (*PD-L1*) gene [112,113,114,115,116].

There is a predilection for the proximal stomach or gastric stump and the male sex. The prognosis of EBV-associated GC is better than the non-EBV-associated counterparts. Treatment is surgical resection in the early stage, and advanced-stage GC may require DNA methylation inhibitors, proteasome inhibitors, histone deacetylase inhibitors, EBNA1 inhibitors, and EBV-specific cytotoxic T-cell infusion [112,117].

##### Lymphoepithelial Carcinoma of the Lung (LEC)

LEC is classified as a poorly differentiated SCC in the WHO Classification [118]. The carcinoma accounts for <1% of non-small cell lung cancer (NSCLC) and predominantly affects younger (median age 51 years) female Asian non-smokers [118,119]. EBV association is >90% in Asian patients but much less in Europeans. EBV serology titer is correlated with burden and stage [120]. LEC does not harbor the genetic alterations common to conventional NSCLC. Histologically, there is a resemblance to NKSCC of the NP, often with pushing borders (Figure 2D–F). It is therefore important to exclude secondary from NP NKSCC. Survival is better compared to other NSCLCs. Treatment is surgical resection, with neoadjuvant or adjuvant chemotherapy, chemoradiotherapy, or immunotherapy in advanced disease [120].

##### Thymus Epithelial Tumors (TETs)

TETs encompass thymomas and thymic carcinomas. Despite isolated reports of EBV association in thymomas, the involvement of EBV in thymomas remains controversial [121,122,123]. EBV etiological involvement in thymomas is not recognized in the WHO Classification. The WHO and others recognize LEC to be EBV-associated [121,122,124]. About half is etiologically related to EBV. EBV is almost always positive in children and young adults and is only uncommon in those >30 years of age [124]. It is a poorly differentiated SCC with a rich lymphoplasmacytic infiltrate, similar to NP NKSCC and lung LEC. Lymphocyte-poor cases are recognized, and diagnosis is supported by EBER-positivity. Thymic LEC accounts for 1–6% of thymic carcinomas and affects more males with a median age of 41 years (range 4–76 years) [124,125]. Most advanced cases present with superior vena cava syndrome. There is no association with myasthenia gravis. LEC spreads locally to neighboring organs. Lung, liver, and bone are the more frequent metastatic sites [123]. The prognosis is poor, which is independent of EBV status [124].

##### Burkitt’s Lymphoma (BL)

The role of EBV in BL has recently been elucidated. The defining molecular event in BL is the juxtaposition of the *myc* oncogene to the immunoglobulin gene heavy chain (*IGH*) locus t(8, 14)(q24; q32) and less commonly to the IG lambda (*IGL*) or kappa (*IGK*) locus t(8, 22)(q24; q11) or t(2, 8)(p12; q24). This translocation activates the *myc* gene, leading to lymphomagenesis [126]. EBV infection in BL contributes to suppression of *myc*-induced apoptosis through the latency proteins EBNA1, EBNA2, ENA3A, EBNA3C, LMP1, LMP2A, the latency microRNAs BARTs, EBERs, and the lytic proteins BHRF1, BALF1 [97,102].

EBV is associated with almost 100% of BL in endemic areas (sub-Saharan Africa, Malawi, Uganda, Cameroon) but shows much lower association (20%) in other areas (sporadic BL) or immunodeficiency-associated BL [127]. BL may be typed as EBV-positive and EBV-negative, with differing biology and pathogenesis. EBV-negative BL features a higher frequency of mutations in *TCF3* and its negative regulator *ID3*, resulting in tonic B-cell receptor (BCR) signaling. In EBV-positive BL, intense antigenic pressure induced by EBV causes TME interactions on BCR, leading to BCR chronic stimulation, B-cell clonal expansion, and neoplasia [128,129]. Other cofactors in BL oncogenesis are coinfection with malaria or HIV [99,130,131]. EBV-negative BL, however, may be the result of a “hit-and-run” initial EBV infection that plays an initiating role to be followed by loss of the EBV genome on acquisition of more stable genetic and epigenetic status in the lymphoma cells [132].

**Figure 3:**
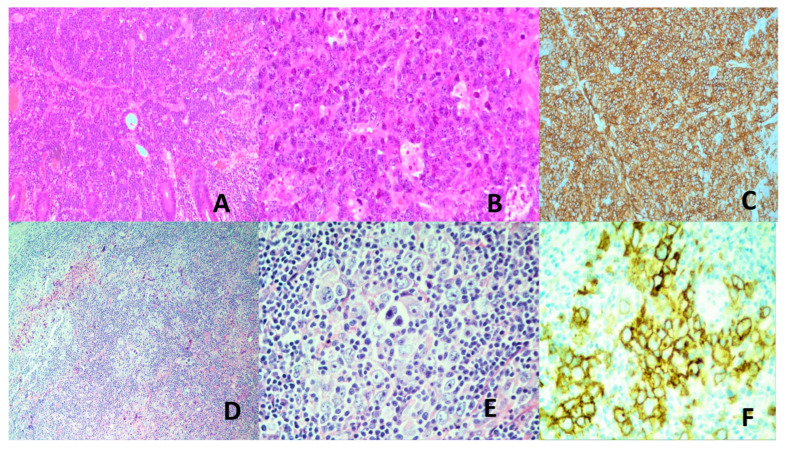
(**A**–**C**) Burkitt Lymphoma. M/74, jejunal tumor. (**A**) Mural invasion, H&E ×100. (**B**) Medium-sized “squared” tumor cells and “starry sky” appearance, H&E ×400. (**C**) CD20-positive ×100. (**D**–**F**) Classic Hodgkin Lymphoma, mixed cellularity type. M/41, large cervical lymph node. (**D**,**E**) Hodgkin and Reed–Sternberg cells in mixed cell backdrop, H&E ×100 (**D**) and ×400 (**E**). (**F**) CD30 highlights neoplastic cells ×200.

BL is characterized histologically by invasive sheets of immunophenotypically B lymphoma cells with squared off cytoplasmic borders, round nuclei, fine clumped chromatin, brisk mitotic activity, often with interspersed tangible body histiocytes to impart the characteristic “starry sky” appearance (Figure 3A–C) [126,133]. Employing contemporary immunochemotherapy, prognosis is good with overall survival of 90% in children and 80% in adults [126,133,134].

##### Hodgkin Lymphoma (HL)

HL is classified into classic HL (CHL) and nodular lymphocyte predominant HL (NLPHL). The neoplastic cells in CHL are the Hodgkin and Reed–Sternberg (HRS) cells, while those in NLPHL are germinal center-derived B-cells known as LP (lymphocyte predominant) cells [135]. CHL is usually EBV-associated, while NLPHL is not. The neoplastic HRS cells are crippled germinal center (GC) cells plagued with high loads of somatic mutations in the rearranged *IGH* gene. These deleterious mutations usually cause apoptosis. The HRS cells, however, are salvaged by survival signals, including those from EBV infection (vbcl2) [104,136]. HRS cells have mostly lost their B-cell phenotype due to the remarkable genetic and metabolic aberrancies. The mixed cellularity and lymphocyte-depleted CHL subtypes are most frequently EBV-positive, while the nodular sclerosing type is mostly EBV-negative [135]. The histopathology of CHL varies with the subtypes, but all show HRS cells in variable amounts with a TME of reactive lymphocytes, eosinophils, histiocytes, neutrophils, plasma cells, and fibrosis in varying proportions according to the subtype [135]. CD30 and CD15 immunopositivity is frequent in the HRS cells (Figure 3D–F). A great majority of CHL can be cured with modern polychemotherapy and radiotherapy [135].

##### Extranodal NK/T-Cell Lymphoma (ENNKTL)

**Figure 4:**
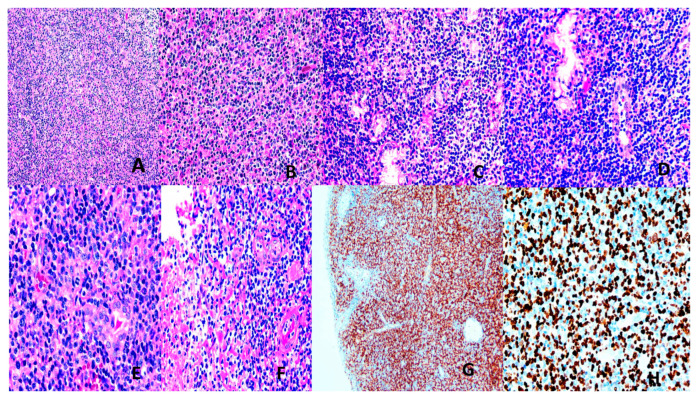
ENNKTL of the nose. M/44, nasal tumor. (**A**,**B**) Mixed lymphoid infiltrates, H&E ×100 (**A**), ×200 (**B**). (**C**–**E**) Glandular epithelium invasion, H&E ×100 (**C**), ×200 (**D**,**E**). (**F**) Vascular invasion with necrosis, H&E ×200. (**G**) CD56 positivity ×100. (**H**) EBER-positive tumor cells x100.

Most (60–90%) ENNKTL are derived from NK cells, and the remaining 10–40% from T-cells [7,137]. Eighty percent are nasal and 20% non-nasal. Nasal NNKTL presents with midfacial disfiguring, destructive lesions of the upper aerodigestive tract, and affects males in the 4th to 5th decades. It is histologically characterized by diffuse sheets of atypical lymphoid cells, usually with a broad range of sizes and irregular nuclei. Epithelial invasion, angiocentricity, angioinvasion, and extensive geographical necrosis are typical features. The NK-cell type is negative for surface CD3, T-cell receptor (TCR), and positive for CD56 with no *TCR* rearrangement. The T-cell type is surface CD3+, TCR+ with rearranged *TCR*. There is frequent cytotoxic molecule expression (Figure 4). A strong etiologic relationship with EBV (latency 2) is present [7,133,137]. A multitude of alterations in genes encoding proteins of the JAK/STAT and NF-KB pathways and epigenetic changes, including dysregulated microRNA, tumor suppressor genes, *myc* overexpression, and effects of EBV latency products (LMP1 contributes to immune evasion) are operative [7,133,137]. Treatment involves concurrent or sequential chemoradiotherapy using L-asparaginase-containing non-anthracycline regimes. Immune checkpoint inhibitors and stem cell transplantation may be required [7,133,138]. Plasma EBV DMA level is an indicator of chemosensitivity and prognosis [7,133,139].

##### EBV+ Nodal T- and NK-Cell Lymphoma (NTNKL)

EBV plays a major etiologic role in EBV+ NTNKL [7,139]. Most display latency 2, though LMP1 is often undetectable. Over 80% is of T-cell lineage, mostly CD8+ with alpha-beta TCR phenotype, though a gamma-delta TCR phenotype with inferior survival may be present. Expression of cytotoxic molecules is frequent. The involved lymph nodes are infiltrated by medium-sized to large blastic tumor cells. Angiocentricity and necrosis are not typical. NTNKL is genomically unstable with alterations in genes related to the JAK/STAT and NF-KB pathways. The degree of genomic instability is lower than ENNKTL. The prognosis is grave [7,140].

##### Systemic EBV+ T-Cell Lymphoma of Childhood (SEBVTCLC)

There is a strong EBV etiologic association related to an undefined genetic defect in the host immune response to EBV [141]. Transformation and progression from systemic chronic active EBV disease (CAEBV) may occur [7]. The involved tissues show infiltration by atypical lymphoid cells of a broad cytologic spectrum. Most cases are CD8+, while those arising from systemic CAEBV are CD4+ [7]. Distinction from fulminant systemic CAEBV and hemophagocytic lymphohistiocytosis is often difficult [7]. SEBVTCLC is highly aggressive with a grave prognosis.

##### Aggressive NK Cell Leukemia (ANKL)

ANKLs are rare and almost always EBV-associated. About 20 cases of EBV-negative ANKL have been reported [7,142]. It is a systemic disease causing hepatosplenomegaly with involvement of the peripheral blood and bone marrow. The atypical leukemia cells may show granular or agranular cytoplasm with a broad spectrum of atypical nuclei. They are CD56+, cytotoxic molecules+, and surface CD3-. Genetically, there are alterations in genes involved in the JAK/STAT and RAS/MAPK pathways. Prognosis is grave with no established treatment protocol [7].

##### EBV+ Inflammatory Follicular Dendritic Cell Sarcoma (IFDCS)

EBV+ IFDCS is rare, with only about 80 reported cases, and is distinct from EBV-negative FDCS. It affects predominantly the spleen and liver, though GIT and upper aerodigestive tract cases have been reported [143]. Asians are mostly affected, with female predominance. The neoplastic cells are spindle to oval, occurring singly or forming loose fascicles and whorls. In IFDCS, there is a prominent lymphoplasmacytic infiltrate, due to which the tumor has been interpreted as an inflammatory pseudotumor [144]. A recent study identified three subtypes of IFDCS: classic, lymphoma-like, and hemangioma-like, thus often causing diagnostic confusion [145]. Surgery is the mainstay treatment, and most patients have favorable outcomes [143,144,145].

##### Other EBV-Associated Lymphoproliferative Diseases (LPDs)

These include EBV-positive mucocutaneous ulcer (MCU), lymphomatoid granulomatosis (LyG), EBV-positive diffuse large B-cell lymphoma (DLBL), and plasmablastic lymphoma (PBL). These conditions are pathogenetically related to immunodeficiency and dysfunction (IDD). In EBV+ DLBL, which affects mostly the elderly, IDD may be due to immunosenescence, which may also account for IDD in LyG. In EBV+ MCU, IDD may be inborn or acquired. IDD in PBL is acquired (as in HIV infection or immunosuppressive therapy) or related to immunosenescence. IDD causes an abnormal host response to EBV. EBV+ MCU affects the mucosal and cutaneous tissue only, while LyG affects the lungs predominantly. Both MCU and LyG show invasion by polymorphous atypical lymphoid cells with frequent angioinvasion and necrosis (in high-grade LyG). EBV+ DLBL may be nodal or extranodal with polymorphic or monomorphic lymphoid infiltrates dominated by blastic cells exhibiting the activated B-cell immunophenotype (IRF4/MUM1+). EBV+ MCU may regress spontaneously after correction of IDD. The prognosis of LyG and EBV+ DLBL is variable. PBL is prognostically poor [146,147,148,149].

#### 3.2.3. EBV Vaccines

Considering the multitude of cancers etiologically and pathogenetically related to EBV infection, prevention of EBV infection is theoretically effective in preventing a multitude of EBV-related cancers [13]. Throughout the past decades, much effort has been made to develop prophylactic EBV vaccines, albeit with limited success. The following is a summary of the current state of EBV vaccine development.

##### Prophylactic Vaccine

EBV infects primarily B-cells and epithelial cells. The envelope proteins gH/gL, gB (the core fusion machinery), and gp350 bind to CD21 on the B-cell surface, followed by cell entry. Entry of EBV into epithelial cells involves binding of EBV BMRF2 to epithelial cell integrins, followed by gH/gL and gB binding. To prevent EBV infection, these envelope proteins pose obvious targets for vaccine development [13,91,96,150]. Early efforts have focused on gp350, which is the most abundant envelope glycoprotein of the virus. The anti-gp350 vaccine, however, did not protect against EBV infection, probably reflecting that gp350 is not strictly required for EBV cell entry [13,96,150,151]. Subsequent vaccines developed against the core fusion machinery proteins gH/gL and gB induce markedly higher EBV neutralizing antibodies compared to gp350 in humanized mice [150]. gH/gL and gB packed in nanoparticles or presented as VLP, possibly through inducing high titers of neutralizing antibodies, may disrupt native conformational epitopes of the EBV envelope proteins. The antibodies induced by these vaccine platforms are quantitatively, though not necessarily qualitatively, robust [150]. Recombinant vaccines express native epitopes and may also elicit high antibody responses both quantitatively and qualitatively, and are potentially ideal for EBV prophylactic vaccination [150,152]. The use of multiple cell surface receptors by EBV to establish an infection adds to the difficulty of developing an effective vaccine [99]. Despite success in humanized mice [150], further trials in human subjects are warranted for safe, efficacious use. A Phase 1 clinical trial comprising mRNA coding gH, gL, g42, and g220 glycoproteins has been initiated and has resulted in increased antibody titers and decreased EBV copy numbers in the enrolled subjects. It is also important to evaluate the durability of the elicited humoral and cellular immune responses [153,154]. As EBV is a ubiquitous virus infecting over 90% of the world population, there is also the logistical difficulty of identifying target age groups and populations to be vaccinated [7,13]. It is likely that EBV vaccine efficacy trials will focus on prevention of infectious mononucleosis in college students or military recruits in high-resource settings [155,156]. There is, to date, no approved effective prophylactic vaccine for EBV infection.

##### Therapeutic Vaccine

A Phase 1 trial for a peptide vaccine against LMP2 and a Phase 2 trial for a viral vectored vaccine against EBNA1/LMP2 for NPC have been completed. There is also an ongoing mRNA vaccine trial for NPC [154] (Table 3). No therapeutic vaccine has been developed for EBV-driven cancers.

#### 3.2.4. Viral Coinfections

##### EBV and HPV

The interplay of this coinfection in uterine cervical intraepithelial neoplasia and SCC has been discussed under Section 3.1.4.

##### Coinfection of EBV and Kaposi Sarcoma-Associated Herpes Virus (KSHV)

This will be discussed in the following sections.

##### EBV and HIV

HIV coinfection dampens the host immune response against EBV, thus aggravating the cancer severity and incidence. EBV+ BL and HL occur more frequently in HIV co-infected subjects. The inflammatory microenvironment and the HIV antigens promote the growth of EBV-associated lymphoma cells. This repertoire induces increased mutation rate and *myc* translocation (in BL) [91,99].

### 3.3. Kaposi Sarcoma-Associated Herpes Virus (KSHV)

#### 3.3.1. Virology and Oncogenesis

KSHV, also known as human herpes virus 8 (HHV8), was identified as herpes-like DNA sequences in HIV-associated Kaposi sarcoma [157]. It is a gamma 2 herpesvirus, which, unlike its widely prevalent counterpart EBV, occurs with a much lower worldwide incidence of 2–5%. It is prevalent in endemic areas of sub-Saharan Africa and the Mediterranean [158]. KSHV is transmitted by saliva primarily in childhood, or through sex (especially men with men sex). Coinfection with HIV and EBV may occur. Control of HIV with antiretrovirals reduces the prevalence of HIV-associated KS [159]. Host cell infection is by interaction of the heparin sulfate receptor with the viral K8.1 envelope glycoprotein [158]. KSHV produces both lytic and latent infections, both of which are important for persistent infection and oncogenesis [160]. KSHV is etiologically associated with KS, primary effusion lymphoma (PEL), multicentric Castleman disease (MCCD), KSHV-positive DLBL, and KSHV-positive germinotropic lymphoproliferative disorder [99,158,160,161,162,163,164,165,166,167,168,169,170]. The latent KSHV gene oncogenic products are latency-associated nuclear antigen (LANA), viral (v) cyclin, vFLIP, Kaposin A, B, and C, microRNA, and ORFK1. LANA dysregulates cell growth and survival, and contributes to viral persistence and immune evasion. There are oncogenic lytic products related to inflammation (vGPCR, vIL6, K15), angiogenesis (vIL6, vGPCR, vCCL1, vCCL2), cell growth (vIL6, vGPCR, K1), and apoptosis inhibition (vCCL1, vCCL2, vbcl2, vIRF1, K1) [158,160]. The virus also causes expression of immunoregulatory genes that hinder host innate and adaptive anti-viral responses [171,172,173]. KSHV affects endothelial cells or their progenitors, conferring them characteristics of lymphatic endothelium [174]. The worldwide incidence of KSHV exceeds that of KS, and factors other than the virus may be at play in oncogenesis. Host chronic inflammation and immune dysfunction play an important part in oncogenesis and tumor progression [3,163,171,172,173].

#### 3.3.2. KSHV-Associated Cancers

The non-neoplastic KSHV-associated MCCD and KSHV-positive germinotropic lymphoproliferative disease (LPD) [156] will not be discussed below, though these may progress to B-cell lymphoma.

##### Kaposi Sarcoma (KS)

KS is a vascular neoplasm of intermediate malignant potential and behavior, varying from indolent to locally aggressive. It is named after Moritz Kaposi, who first described the disease as “idiopathic multiple pigmented sarcoma of the skin” in 1872 [175]. KS affects the skin, lymph nodes, or other viscera (lungs, GIT). Involved sites vary with the epidemiologic type of KS: classical (skin), endemic (lymph nodes in children, skin in adults), transplant-related, and HIV epidemic (skin, mucosa, viscera) [159].

**Figure 5:**
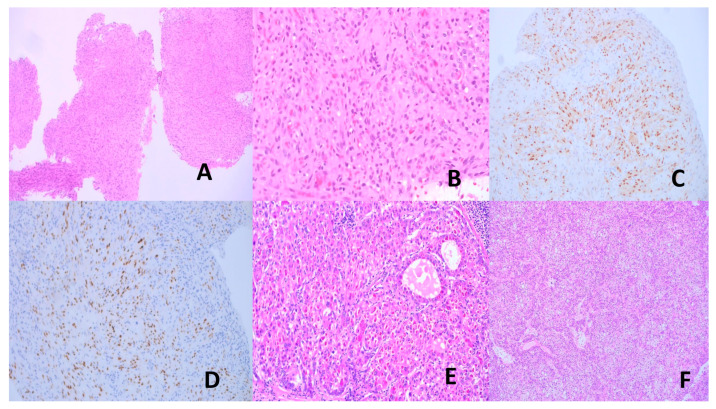
(**A**–**D**) Kaposi sarcoma. M/38, HIV-positive, multiple skin lesions. (**A**) Spindle tumor cells, H&E ×100. (**B**) Red cells in slits and spaces among spindle tumor cells, H&E ×400. (**C**) ERG-positive tumor cells ×100 (**D**) LANA-positive tumor cells ×100. (**E**,**F**) Hepatocellular carcinoma. M/61, liver tumor. (**E**) Pseudoglandular pattern and many extracellular globules, H&E ×200. (**F**) Macrotrabecular pattern and fatty change in tumor cells, H&E ×200.

Lymph nodes may be primarily involved or secondarily involved when draining a nearby KS. The early lesion occurs in the nodal capsule, with extension along fibrous trabeculae of the lymph node. The early lesion is subtle and difficult to recognize. In the tumoral stage, there are interlacing fascicles of deceptively bland-looking mitotically inactive spindle cells forming a sieve-like pattern with erythrocyte-filled vascular spaces. The tumor cells express an endothelial cell immunophenotype. The diagnostic marker is LANA detectable by immunohistochemistry [158,160] (Figure 5A–D). LANA staining is preferred over polymerase chain reaction for viral DNA, as the associated inflammatory cells may also harbor the virus [171]. Similar histological features are found in other involved organs.

There is at present no curative treatment for KS [164]. Optimal control of HIV with ART alleviates severity. In iatrogenic KS, reduction of immunosuppressive therapy is helpful. Surgery may be indicated for cosmetic reasons. Targeting endothelial cells may be the future therapeutic direction [163,164,172].

##### Primary Effusion Lymphoma (PEL)

PEL is a large B-cell lymphoma presenting as lymphomatous effusions and rarely as body cavity-related tumor masses. Most are HIV-positive with frequent EBV coinfection [99,165,166,167,176,177]. There is often co-occurrence of other KSHV-associated diseases (KS and MCCD) in 50% of cases [166,167]. PEL can occur in the elderly without HIV infection and may be related to immunosenescence [168,169,170]. The tumor cells are blastic or anaplastic among apoptotic debris [167,169]. Demonstration of LANA is essential for diagnosis. PEL is aggressive with a poor prognosis. EBV-associated PEL may show better outcomes [170].

##### KSHV-Positive Diffuse Large B-Cell Lymphoma (DLBL)

KSHV+ DLBL are rare and occur in a backdrop of severe immunodeficiency involving lymph nodes and/or spleen with possible extranodal and peripheral blood dissemination. It affects mostly HIV+ men aged 30–40 years. There is infiltration by sheets of blastic B-cells positive for LANA. It is extremely aggressive with a poor prognosis [178].

#### 3.3.3. KSHV Vaccines

KSHV vaccines target KSHV structural glycoproteins that are important for host cell attachment, fusion, and entry. These are K8.1, gB, gH, gL, gM, and gN [14,179]. K8.1. gB and gH/gL have been used in VLP-based vaccines and resulted in neutralizing antibody responses in mice and rabbits that prevented in vitro infection of epithelial, endothelial, fibroblastic, and B cells [14,180,181]. Other KSHV antigen targets include non-structural proteins unique to the virus. LANA is a maintenance protein expressed in all KSHV-infected cells and is an obvious target for vaccine development. However, LANA is an intracellular antigen and is unlikely to be targeted by humoral immunity. It is therefore a good target candidate for mRNA vaccines for eliciting cellular immunity (CI) when the antigen is modified to eliminate its CI-escape motif [182]. There are several other viral surface-expressed non-structural proteins that may be targeted. They are K1 protein, v!L6, G protein-coupled receptor, and viral chemokines [14]. Short of a dominant viral antigen target, the myriad structural and non-structural viral proteins suggest that vaccines developed against multiple viral antigens are the direction to follow [14]. Though successful immunogenicity is demonstrated in small animal models with vaccines against structural and surface proteins, there is hitherto no approved effective vaccine against KSHV for humans [14,180,181].

#### 3.3.4. Viral Coinfections

##### KSHV and HIV

This is a common occurrence in KS, KSHK+ PEL, and DLBL, as discussed above.

##### KSHV and EBV

This may be observed in PEL, where the outcome may be better [170]. It may also be possible that the viruses are supportive of each other [176,183,184].

##### KSHV, HIV, and EBV

This may occur in KSHV+ DLBL, where EBV occurs in 80% of cases. Coinfection with EBV in HIV+ KS of the conjunctiva has also been reported [185].

### 3.4. Human Immunodeficiency Virus (HIV)

#### 3.4.1. Virology and Role in Oncogenesis

HIV is an RNA retrovirus belonging to the subgroup lentivirus [186,187]. It came to light after reports of KS and pneumocystis pneumonia among men who had sex with men (MSM) in 1981 [188]. HIV targets active CD4+ cells and macrophages. Most of the initial viral replication occurs in lymph nodes [186]. HIV possesses an envelope containing two glycoproteins gp41 (transmembrane) and gp120 (surface) [186,187]. The viral capsid is constituted by p24, which encloses two viral RNA strands of the viral genome. Gp16, a matrix protein, is anchored to the internal surface of the envelope. p9 is a nucleocapsid protein not covalently attached to the viral RNA. There are six regulatory proteins: tat, reverse transcriptase (RT), Nef, Vif, Vpu, and Vpr, which are essential for viral replication. HIV enters lymphocytes and monocytes through viral protein gp120 cognate recognition of the CD4 molecule and chemokine receptor (CXCR4/CCR5). This is followed by fusion between the viral envelope and the host cell membrane, resulting in the release of the viral capsid into the cell cytoplasm [186,187]. Though HIV does not replicate in B-cells, it produces severe B-cell dysfunction [187].

HIV has not been identified to cause a specific cancer. The role of HIV in oncogenesis is mostly through immune dysfunction, persistent immune activation, and chronic inflammation caused by the infection. Coinfection with HIV and other oncogenic viruses positively contributes to cancer development [82,99,189,190]. There is, however, evidence that some HIV proteins may be directly involved in oncogenesis. The five HIV proteins that may be oncogenic are tat, Nef, gp120, p17, and RT. They contribute to oxidative stress, enhanced EMT, decreased tumor suppressor gene expression, glycolysis stimulation, cell proliferation, migration, survival, angiogenesis, and immunomodulation [82,190].

#### 3.4.2. HIV Vaccines

Identification of the various viral proteins and their roles in host cell infection has spawned much research in HIV vaccine development. More than 20 candidate vaccines developed against the proteins gp120, gp160, multimeric gp120, and V3 peptide have been tried in human subjects, including prophylactic and therapeutic vaccines [187]. However, there are hitherto no approved effective HIV vaccines, which may be due to rapid mutation of the virus, integration of viral DNA into the host DNA, and effective immune evasion [191].

#### 3.4.3. Viral Coinfections

Coinfection of HIV with the oncogenic viruses HPV, EBV, and KSHV results in increased incidence and severity of the cancers caused, as discussed above. The role of HIV coinfection in oncogenesis induced by the hepatitis viruses B and C will be discussed below.

### 3.5. Hepatitis Viruses

#### 3.5.1. Virology and Oncogenesis

##### Hepatitis B (HBV)

HBV is a partial double-stranded DNA virus belonging to the Hepadnaviridae family. There are eight genotypes A to H. It consists of one complete coding minus (−) strand and one incomplete non-coding plus (+) strand. There is encapsulation by a lipoprotein membrane constituted by three hepatitis B surface antigens (HBsAg): large, middle, and small forms. The nucleocapsid is composed of the hepatitis B core protein (HBc), viral polymerase (pol), and the viral genome DNA. Entry into the host cell entails low specificity binding between HBsAg and heparin sulfate proteoglycans on the hepatocytic surface, followed by high-affinity interaction between the HBV envelope and sodium taurocholate co-transporting polypeptide receptor on hepatocytes, endocytosis of the virion, and release of relaxed circular DNA (rcDNA) into the hepatocytic cytoplasm. rcDNA is converted into covalently closed circular DNA (cccDNA), which encodes for RNA and is then translated to produce structural and non-structural viral proteins. Persistence and stability of cccDNA are key to HBV chronic infection. cccDNA also transcribes to pregenomic RNA (pgRNA), which is reverse transcribed to double-stranded linear DNA (dlsDNA). The latter is responsible for the integration of the viral genome into host DNA, which is important for hepatocytic damage and carcinogenesis. While viral integration was believed to be random, there is recent evidence that host cancer-associated gene segments may be favored sites for integration. These are fragile chromosomal sites containing HBV preferred CpG islands. These sites are *TERT*, *KMT2B*, *CCNE1*, *PAK3*, *CCND1*, and *FGF19*. *TERT* appears to be most favored for viral integration, leading to overexpression of telomerase, conservation of telomere length, and thus inhibition of cell senescence and tumor cell growth promotion [192,193,194,195]. Viral integration also causes chromosomal instability, modulation of host oncogenes and signaling pathways, expression of truncated HBV mutant proteins (such as hepatitis B virus X protein—HBX), inhibition of apoptosis, induction of stemness, and host immune dysfunction [192,193,194,195]. Recent scRNA-seq studies have enlightened the relationship between tumor heterogeneity and immune cells in the tumor microenvironment [195].

HBV is a class I carcinogen and a major cause of hepatocellular carcinoma (HCC) [196]. HCC ranks fifth in global cancer incidence and is third in leading causes of cancer deaths [192,197].

##### Hepatitis C (HCV)

HCV is an enveloped positive-stranded RNA virus belonging to the Flaviviridae family. It consists of the structural proteins E1 and E2, and non-structural proteins P7, NS2, NS3, NS4A, NS4B, NS5A, and NS5B. There is extraordinary genetic diversity with seven genotypes and more than 80 subtypes [198]. It is a major cause of HCC, and may also cause cancers of the oral cavity, oropharynx, intrahepatic bile ducts, pancreas, kidney, and B-cell lymphoma [199,200]. The causation of B-cell lymphoma may be related to sustained antigen stimulation leading to oligoclonal or monoclonal B-cell expansion (in marginal zone lymphoma), direct infection of B-cells (in DLBL), and transformation to IgHBCL2 B-cell clones due to inflammatory and cytokine stimulation (in follicular lymphoma) [200]. Unlike HBV, HCV cannot integrate into host cell DNA. It causes cancer through direct interaction of viral proteins with the host immune system, tumor suppressor genes, and oncogenes [199,201,202]. This results in persistent cellular proliferation, promotion of stemness, inhibition of apoptosis, angiogenesis, and an inflammatory milieu. This involves promoting tyrosine kinase receptor signaling, Hedgehog signaling, NF-KB and STAT3 signaling, p53 and pRB tumor suppressor protein cytoplasmic retention, and EMT promotion [199].

#### 3.5.2. Hepatitis Virus—Associated Cancers

HBV and BCV are major causes of HCC. Other cancers that can be caused by HCV will not be covered in this section. HCC accounts for 75–85% of primary liver cancers, being the 6th most common cancer and the 4th leading cause of cancer deaths worldwide. It is more common in Africa, China, and Southeast Asia [203]. HCC develops in a backdrop of chronic liver disease, which is often also caused by the hepatitis viruses. Other HCC predisposing chronic liver diseases and conditions are chronic alcoholism, exogenous fungal toxins, steatohepatitis, and inherited metabolic diseases [204,205]. HCC can be multifocal and spreads by lymphatic and hematogenous routes [205]. Histologically, HCC is characterized by hepatocytic differentiation with variable degrees of cytologic atypia, increased arterialization and sinusoidal capillarization, and varying trabecular, solid, pseudoglandular, macrotrabecular, and mixed patterns (Figure 5E–F). Some HCC subtypes are distinct clinicopathologic and molecular entities: fibrolamellar, schirrhous, clear cell, steatohepatitic, massive macrotrabecular, chromophobe, neutrophil-rich, and lymphocyte-rich. Histologic grading is by degree of likeness to normal hepatocytes. However, a rigorously defined, reproducible, and easy-to-use grading system is yet to be defined [205,206]. The prognosis of HCC is poor, especially in advanced disease [205].

#### 3.5.3. Hepatitis Virus Vaccines

##### HBV

Several Food and Drug Administration (FDA) approved multimeric particulate anti-HBV vaccines are available. These are prophylactic neutralizing recombinant vaccines developed against the HBsAg, specifically against the major hydrophilic region (MHR) containing all three forms of HBsAg. They provide protection against all HBV genotypes (A to H). However, alterations of residues within the MHR can result in replication of the mutated virus, which would evade protection by the vaccines [207,208]. The FDA approved single vaccines, including Engerix-B, Recombivax HB, HEPLISAV-B, and PreHevbrio (Table 2). There are three approved combination vaccines: Pediatrix, Twinrix, and Vaxelis, where vaccines against other infections are combined. HBV vaccines are used in universal vaccination programs at birth in many parts of the world. This resulted in a profound reduction of HBV infections, HBV-related chronic disease, and HCC [11,196,209,210,211,212,213].

##### HCV

Despite knowledge and understanding of the structural envelope, non-structural, and core proteins of the virus, no approved effective anti-HCV vaccines are available to date. This may be related to a lack of high replication of HCV in non-human primate cell lines, which impairs the development of live attenuated vaccines, the great virus genetic diversity and subtype variability, poor understanding of the host immune response to HCV, and difficulty in recruiting study cohorts due to social, psychological, and marginalization issues [198,214]. Vaccines using recombinant virus vector or VLP platforms against structural and non-structural proteins have been studied without success [198,214]. A minicircle-based vaccine against chimeric HBV-HCV VLP has been reported to induce potent T-cell and antibody responses against HCV in BALB/c mice [215]. The introduction of highly effective anti-viral drugs against HCV has very likely diminished commercial interest in anti-HCV vaccine development [201].

#### 3.5.4. Viral Coinfections

##### HBV and HIV or HCV and HIV

Apart from HIV-induced immunodeficiency causing decreased clearance of HBV or HCV from the liver, HIV may contribute to oncogenesis through increased liver damage, inflammation, and fibrosis. HIV infection results in elevated levels of circulating lipopolysaccharides (LPS) in the portal and systemic circulation. LPS activates hepatic stellate cells (HSCs) and Kupffer cells (KFCs), thus inducing oxidative damage, proinflammatory cytokine release, and fibrosis. HIV can directly infect HSCs and KFCs, leading to similar pro-oncogenic results. In HIV/HCV coinfected individuals, HCC occurs at a younger age, with the risk of HCC development being increased by 11% each year compared to HIV-negative individuals [82,188,216,217].

##### HBV and HCV

Coinfections of HBV and HCV increase the risk of HCC compared to the mono-infections. The coinfections cause greater progression to advanced chronic liver disease and HCC. This may be related to long chronic disease duration, high levels of liver fibrosis, and carbohydrate intolerance [218,219]. HCV dominance is more common than HBV dominance in the coinfection, which is relevant to treatment protocols [220].

##### HBV, HCV, and HIV

Coinfections of HBV, HCV, and HIV carry higher risks of HCC development compared to HBV or HCV mono-infections. The triple infection, however, carries a lower rate of HCC compared to coinfections with HCV and HIV [221].

### 3.6. The Merkel Cell Polyoma Virus (McPyV)

#### 3.6.1. Virology and Oncogenesis

The McPyV is the only oncogenic virus among the 14 human polyomaviruses. It was discovered in 2008 [222,223,224,225] and is a small non-enveloped DNA virus. There is a circular double-stranded genome of approximately 5400 base pairs (bp) [223,224]. It contains an early region which encodes the oncoproteins large T (LT), small T (sT), and the 57kT protein. The role of the 57kT protein is still nebulous. The late region of the viral genome encodes the capsid structural proteins VP1, VP2, VP3, and microRNAs [223,224,226]. McPyV enters the host cell through attachment of VP1 to sulfated glycosaminoglycans (particularly heparin sulfate), followed by attachment to sialylated glycan co-receptor for gene transduction. Viral integration is important for oncogenesis. This results in mutations leading to the truncation of LT [223,224,225]. The truncated LT is capable of binding to and inactivating the Rb protein [223,225,226]. The sT induces hyperphosphorylation of the cellular translation factor 4E-BP1 in an mTOR-dependent manner, causing cell cycle progression and transformation [223,225,226].

#### 3.6.2. The McPyV-Associated Cancers

The major McPyV-associated cancer is Merkel Cell Carcinoma (MCC), though other malignancies, including skin SCC, basal cell carcinoma, melanoma, and cutaneous B- and T-cell lymphomas have also been related to McPyV [224]. Though McPyV is named after Merkel cells, the cell origin of McPyV-associated MCC is debatable [226]. Other possible histogenetic origins, including epidermal progenitor cells, pre-/pro-B-cells, and dermal fibroblasts, have been proposed [224,226]. MCC is a rare, highly aggressive primary cancer of the skin affecting the dermis and subcutis in large tumors. The epidermis may be the only involved site, and pure epidermal MCC has been described in McPyV-negative cases [226,227,228]. There are invasive sheets of small round cells which are positive for CK20 and neuroendocrine markers [224,228]. The risk factors include old age, sun exposure, white skin, male sex, immunosuppression, and history of skin malignancies [224,227,228]. About 80% of MCC is McPyV-positive, with the remaining McPyV-negative cases related to UV exposure or chronic arsenicism. McPyV-negative MCC exhibits more frequent mutations and poorer outcomes [224,225,226,227]. There is a higher association of McPyV-negativity in MCC combined with other types of skin cancers [228].

#### 3.6.3. McPyV Vaccines

Despite knowledge of the McPyV capsid structural proteins V1, V2, and V3 and the oncoproteins Lt and sT, no effective approved anti-McPyV vaccine has become available [229]. A prophylactic vaccine against the LT antigen has been found to be effective in C57BL/6 mice [230]. Therapeutic vaccines targeted to enhance host immunity and augment anti-tumor/anti-viral effects in the TME have been the focus of research in McPyV-positive MCC. The viral targets used are mostly LT and, more recently, the capsid protein V1. Increased CD4/CD8 cellular responses have been demonstrated using various platforms (recombinant protein, mRNA, DNA, and oncolytic viruses) [229,230,231,232,233]. In addition, potent antibodies against the V1 protein were demonstrated in one study [229]. However, most of the work was performed on experimental animals (BALB/c mice) [229,230,231,232,233]. There was a preclinical in vitro human vaccination study that showed specific killing of MCC cells [230].

#### 3.6.4. Viral Coinfections

Coinfection with HIV interplays with McPyV in oncogenesis. The incidence of MCC in HIV infected individuals is 13 times higher than in the general population [227,234,235]. MCC is also more frequent in immunocompromised subjects (organ transplant or B-cell malignancy patients), and this may also be operative in HIV-infected individuals [227,234,235].

### 3.7. The Human T-Cell Leukemia Virus Type-1 (HTLV-1)/Human T-lymphotropic Virus

#### 3.7.1. Virology and Oncogenesis

HTLV-1 is a retrovirus belonging to the family Retroviridiae and genus Deltaretrovirus [236,237,238,239,240,241]. It is spherical, measures 100–120 nm, and has the proteins gp21 and gp46 making up the envelope. The capsid is constituted by the proteins p15, p19, and p24, which enclose the viral genome. The latter is made up of two identical single-stranded RNAs with protease (p10), RT (p55), integrase (p32), and RNAase. The viral genome encodes the structural proteins (envelope and capsid) and the regulatory proteins Tax and HBZ (HTLV-1 basic leucine zipper factor). The regulatory proteins contribute to the control of cell proliferation and immune–inflammatory responses [236,237,238,239,240,241]. The viral genome is integrated into the host genome as the provirus, facilitated by integrase. The integration occurs more frequently near some “hotspots” [240]. HTLV-1 infects mostly CD4+ T-cells, though CD8+ lymphocytes, dendritic cells, and macrophages may also be infected [236,240]. The infected cells do not undergo lysis, and the proviral genome remains latently integrated, achieving host immune evasion [236,237,238,239,240]. HTLV-1 has six reported subtypes A to F, with most infections caused by subtype A. There are three other HTLV viruses: HTLV-2, HTLV-3, and HTLV-4, but only HTLV-1 is convincingly related to human diseases [236,237,238,239].

Oncogenesis is related to two factors: duration of infection and a high proviral load (PVL). PVL is the percentage of peripheral blood mononuclear cells infected by HTLV-1. The infected cell clones pass through many mitotic events, especially associated with a long infection duration, and become plagued with replication errors and mutations. Some of these replicative errors are deleterious and lead to oncogenesis [240]. Recurrent somatic mutations detected in adult T-cell leukemia/lymphoma involve genes encoding proteins of the T-cell signaling pathway NF-KB: *PRKBB*, *PLCGL1*, *VAV*, and *CARD11*. Mutations in transcription factor encoding genes *IRF4*, *GATA3*, *IKZF2*, and chemoreceptor genes *CCR4*, *CCR7*, and *GPR183* are also implicated [237,240]. A modulated host immune response is also contributory [240].

The HTLV-1 regulatory proteins Tax and NZB contribute by causing genomic instability and conferring survival or proliferative advantage to the infected clones. Tax expression is, however, frequently lost in 50% of Adult T-cell leukemia/lymphoma (ATL) clones and may thus be contributory only in the early stages of oncogenesis. HBZ, on the other hand, is retained in all ATL clones and may be a good target for vaccine development [240].

#### 3.7.2. HTLV-1-Associated Cancer

HTLV-1 is prevalent in Japan, Africa, the Caribbean islands, and Central and South America [239]. The global distribution of HTLV-1 infection is estimated to be 5 to 10 million persons [239,241]. Transmission is predominantly from mother to child through breastfeeding, sexual activity, and blood contact [239]. There are four subtypes of ATL: smouldering, chronic, lymphomatous, and acute. In the most aggressive acute and lymphomatous subtypes, there is lymphadenopathy, hepatosplenomegaly, skin, bone, lung, and multifocal visceral infiltration with hypercalcemia. In the chronic and smouldering subtypes, there are non-specific symptoms and no tumor mass formation. There may be prominent skin involvement. Strongyloidosis is common in all subtypes [239]. The peripheral blood and involved organs show infiltration by markedly pleomorphic medium to large lymphoid cells with marked nuclear abnormalities, causing the so-called “flower cells” or hallmark cells with horseshoe or kidney-shaped nuclei. Immunophenotypically, the cells are CD3+, CD4+, CD25+, CCR4+, and GATA3+ [242]. No effective treatment is available. Inhibition of cell pathway proteins AKT, BET, phosphatidylinositol 3-kinase, and NF-KB has been used with success in mice [240].

#### 3.7.3. HTLV-1 Vaccine

Despite tremendous efforts to develop an effective vaccine, through employing multiple platforms and targeting structural and non-structural proteins of the virus, no effective vaccines have become available [241]. Most studies have been performed on experimental animals. One study on ATL patients using a therapeutic vaccine containing autologous dendritic cells along with Tax peptide and HTLV-1-specific cytotoxic T lymphocytes showed clinical improvement in two patients, with one eventually achieving complete recovery, has been reported [243]. The presence of target populations (pregnant women) in endemic regions may represent a research focus in the future development of prophylactic vaccines [239,240,241].

### 3.8. SARS-CoV-2 Virus

#### 3.8.1. Virology and Possible Oncogenetic Mechanisms

The SARS-CoV-2 virus is a coronavirus belonging to the genus Betacoronvirus. It was responsible for the pandemic COVID-19 (coronavirus infectious disease 2019) and caused infections of hundreds of millions and a death toll of millions of people [244,245]. It is a single-stranded RNA (ssRNA) enveloped zoonotic virus and among the seven coronaviruses that attack humans. The ssRNA encodes structural proteins, including the membrane glycoprotein, envelope protein, nucleocapsid, and the spike (S) proteins. The S protein is responsible for host cell invasion through interaction with the angiotensin-converting enzyme (ACE) on the host cell surface [244,245,246,247]. SARS-CoV-2 infection typically causes multi-organ disease, resulting in respiratory and GI symptoms, and loss of taste and smell [244,245]. COVID-19 may resolve clinically or may result in death due to the severe host immune response and upregulated cytokines and severe inflammation in various organs, especially in the respiratory system [244,245,246]. In convalescence, however, a variety of long-lasting symptoms may persist as the so-called “long COVID-19” [248]. It is theoretically possible that cancer development may occur, as latent viral infection predisposes to oncogenesis [6,7]. A recent report suggests that latent SARS-CoV-2 infection may occur in the testes [249]. There is, however, so far no unequivocal evidence of latency in SARS-CoV-2 infection. Multiple mechanisms of oncogenesis have been postulated for a possible role of SARS-CoV-2. These include activation of oncogenes, inhibition of tumor suppressor activity (nsp3 and nsp15 degrades p53 and pRb), cell cycle and signaling pathways modulation, extracellular vesicles, host genomic instability, epigenetic changes, inflammatory cascade, reactive oxygen species, and EMT [246,247,248,250,251]. There have been reports of SARS-CoV-2 virus infection inducing IL-6 activation of metastatic breast cancer in lungs [252], developing hepatic EBV-negative DLBL [253], and causing progression of KS [254]. However, there are also reports of oncolytic properties of the SARS-CoV-2 virus in various malignancies: NK cell lymphoma, HL, and mycosis fungoides [250,251,255,256,257]. These contradictory reports on the possible oncogenesis of SARS-CoV-2 render further studies on the issue mandatory.

#### 3.8.2. Vaccines

The surprise and rapid onset of the COVID-19 pandemic triggered accelerated development of anti-SARS-CoV-2 vaccines [17]. Various types were developed, and mRNA vaccines have proved to be superior in effectiveness and efficacy. Five years have elapsed since the initial outbreak in China, and many countries have adopted various vaccination strategies and programs against the virus [23,258].

## 4. Developing New Vaccines

### 4.1. Defining Endpoints for Clinical Vaccine Trials

Vaccine trials, like all clinical trials, are conducted in the sequence of preclinical, Phases 1, 2, 3, and 4 studies. Each trial aims to evaluate the candidate vaccine for its safety/tolerability and immunogenicity, with increasing numbers of enrolled human subjects in Phases 1, 2, 3, and 4 trials. Vaccines successful in Phase 3 trials may be licensed by regulatory agencies. Several endpoints of the trials need to be defined: safety endpoints (SEs), immunogenicity endpoints (IEs), and primary endpoints (PEs). SEs range from common side effects to rare adverse events. IEs may include humoral and/or cellular immune response parameters, analyzed separately or with multiplicity adjustment. PEs are particularly problematic for the evaluation of therapeutic vaccines, as oncovirus-attributed cancers develop after long latency infections requiring long-term follow-up of study subjects, rendering the trials difficult and impractical. Setting cancer development as a primary endpoint is also potentially unethical. Surrogate endpoints may be adopted to circumvent these issues, but may pose a problem of their validity in predicting cancer development and progression [259]. A possible surrogate endpoint for EBV therapeutic vaccine trials may be circulating plasma EBV DNA levels.

### 4.2. Clinical Vaccine Trials, Difficulties and Prospects

Despite the accumulated body of knowledge in virology and the mechanisms of virus-attributed cancers, the development of vaccines against many oncoviruses remains disappointing. Apart from the effective approved prophylactic vaccines against HPV and HBV, which have been adopted in universal vaccination programs in many parts of the world with promising results in prevention of the infections and attributed cancers, effective prophylactic vaccines for other oncoviruses and therapeutic vaccines development for human subjects remain disenchanting. This may be related to various factors. Some viruses display multiple strains with genetic diversity [198,214]. Integration of viral DNA into the host genome leads to immune evasion and latent infection [62,191,192,193,194,195]. Some viruses undergo rapid mutation and pose problems in identifying target antigens for vaccine development [191]. The lack of animal models or in vitro systems for immunogenicity evaluation in preclinical studies is another problem encountered [14,99,173,198,205,214]. The use of effective anti-viral drugs may impair commercial interest in vaccine development and research [201]. The adoption of valid surrogate endpoints or realistic disease primary endpoints in clinical vaccine trials [259,260] and the extreme rarity of some virus-attributed cancers are additional hurdles to overcome in clinical vaccine development. Clinical vaccine trials that are cited in papers published in the past 5 years are tabulated in Table 3. Trials performed with only vaccines and no additional immunotherapy or chemoradiotherapy are tabulated. Most were Phases 1 and/or 2 trials, and no results were posted in many of these trials. It is not clear from the posted reports at ClinicalTrials.gov whether further Phase 3 trials would proceed.

## 5. Conclusions

Oncogenic viruses are responsible for 13–15% of human cancers [1,2,3,4,5]. Seven viruses, HPV, EBV, KSHV, HBV, HCV, McPyV, and HTLV-1, are well recognized in causing cancers ranging from carcinoma and sarcoma to lymphoma/leukemia. As oncoviruses are among the known causes of cancer, it is intuitively possible to prevent the cancers caused by developing and using prophylactic vaccines. Recognition of the appropriate target virus antigens/proteins, employment of efficacious vaccine platforms, and effective use of adjuvants are important attributes of successful vaccines. The development and use of prophylactic anti-HPV and anti-HBV vaccines in universal vaccination programs for target at-risk populations have contributed profoundly to preventing the infections (HPV and HBV) and incidence of the cancers caused (cervical and hepatocellular carcinomas). Development of prophylactic vaccines against the other oncogenic viruses has been much less successful. This may be related to less successful recognition of oncogenic viral target antigens/proteins that are instrumental in causing disease, difficulty in defining disease primary endpoints, the validity of surrogate endpoints in clinical vaccine trials, availability of effective anti-viral drugs, and the esoteric nature of some oncovirus-attributed cancers, thus impairing commercial interest in vaccine development. Therapeutic vaccines, though effective in preclinical animal models, remain uniformly unsuccessful in human subjects but may constitute an applicable tool in future cancer treatment. HIV, though probably not oncogenic per se, plays important collaborative roles in oncogenesis in various cancers caused by viruses. Prevention of HIV infection is important in alleviating virus-related cancer incidence and severity in HIV prevalent areas. Regrettably, there are still no effective anti-HIV vaccines. Coinfection by multiple oncogenic viruses is not unusual and contributes to their collaboration and reinforcement in oncogenesis. The role of the SARS-CoV-2 virus in oncogenesis is theoretically plausible. It remains to be seen in long-term follow-up studies on patients with “long COVID-19 disease” whether an oncogenic role of the SARS-CoV-2 virus could be documented.

## Figures and Tables

**Table 1:** Incidence of infection-attributed cancers (2018) [1].

	Incidence	
Infectious Agents	Number of Attributed Cancers	Percentage of Total Infections
*Bacterial Infections*		
Helicobacter pylori	810,000	36.8
Subtotal (Bacteria)	810,000	36.8
*Viral Infections*		
HPV	690,000	31
HBV	360,000	16
HCV	160,000	7
EBV	156,000	7
KSHV	42,000	2
HTLV-1	3600	0.2
Subtotal (Viruses)	1,411,600	63.2
Total Infections	2,221,600	100

Incidence of McPyV-attributed cancers was not included in the statistics [1].

**Table 2:** Oncogenic viruses, cancers, and approved prophylactic vaccines.

*Virus*	*Cancers Caused*	*Prophylactic Vaccines*	*Platform*
HPV	Uterine cervical SCC Vaginal, vulval, penile, anal, oropharyngeal SCC Urinary bladder	Gardasil (bi-,quadri-, nona-valent) Cervarix (bivalent)	Recombinant
HBV	Hepatocellular carcinoma (HCC)	Energix-B Recombivax HB HEPLISAV-B# PREHEVBRIO	Recombinant
		Twinrix *	Recombinant (HBV) Inactivated (HAV)

* combination of Hepatitis A virus (HAV) and Hepatitis B virus (HBV) vaccine.

**Table 3:** Clinical Vaccine Trials cited in papers published 2020–2025.

Virus	Prophylactic (PV) or Therapeutic (TV) Vaccines	Clinical Trials.gov ID Number	Sponsoring Body	Vaccine	Vaccine Platform	Enrolment (Subjects Number)	Trial Phase	Status of Trial
HPV	TV	NCT01266460	Gynecologic Oncology Group	ADXS11-001	Bacterial Vectored	54	2	Completed
	TV	NCT01598792	U of Liverpool	ADXS11-001	Bacterial Vectored	2	1	Completed
	TV	NCT02399813	Advaxis, Inc	ADXS11-001	Bacterial Vectored	36	2	Completed
	TV	NCT02291055	Adaxix, Inc	ADXS11-001	Bacterial Vectored	75	1/2	Completed
	TV	NCT02002182	Baylor College of Medicine	ADXS11-001	Bacterial Vectored	15	2	Completed
	TV	NCT04607850	Barinthus Biotherapeutics	ChAdOx1-hrHPV	Viral Vectored	108	1/2	Completed
	TV	NCT00075569	Albert Einstein College of Medicine	SGN-00101	Peptide	64	2	Completed
	TV	NCT02576561	THEVAX Genetics Vaccine	TVGV-1	Peptide	10	2	Completed
	TV	NCT02405221	Sidney Kimmel Comprehensive Cancer Center, Johns Hopkins	TA-CIN	Peptide	14	1	Completed
	TV	NCT01957878	Genticel	Procervix	Peptide	239	2	Completed
	TV	NCT03821272	U of Arkansas	PepCan	Peptide	17	1/2	Completed
	TV	NCT02481414	U of Arkansas	PepCan	Peptide	81	2	Completed
	TV	NCT03418480	U of Southampton	HARE-40	mRNA	32	1/2	Completed
	TV	NCT01304524	Inovio Pharmaceuticals	VGX-3100	DNA	167	2	Completed
	TV	NCT04131413	Sidney Kimmel Comprehensive Cancer Center, Johns Hopkins	PNGVL4a- CRTE6E7L2	DNA	48	1	Ongoing
	TV	NCT00988559	Sidney Kimmel Comprehensive Cancer Center, Johns Hopkins	PNGVL4a-CRT/E7(Detox)	DNA	132	1	Completed
	TV	NCT03870113	Shenzhen People’s Hospital	DC Vaccine	Dendritic cell-based	80	1	Completed
EBV	PV	NCT05164094	Moderna	mRNA-1189	mRNA	867	1/2	Ongoing
	PV	NCT04645147	National Institute of Health (NIH)	Nanoparticle gp350-ferritin	Nanoparticle	83	1	Ongoing
	PV	NCT06908096	National Institute of Allergy and Infectious Diseases (INIAID)	Nanoparticle gH/gL/g42-ferritin +/− gp350-ferritin	Nanoparticle	750	1	Ongoing
	PV	NCT05831111	NIH	mRNA-1195	mRNA	474	1	Ongoing
	TV	NCT01094405	Chinese U of Hong Kong	EBNA1/LMP2 recombinant vaccine	Recombinant	25	2	Completed
	TV	NCT01147991	Cancer Research UK	EBNA1 c-terminal/LMP2 chimeric protein expressing recombinant + modified Vaccinia Ankara vaccine	Recombinant + Viral vectored	16	1	Completed
	TV	NCT00078494	National Institute of Health Clinical Center (CC)	LMP2:340-349 + LMP2:419–427	Peptide	99	1/2	Completed
	TV	NCT05714748	West China Hospital	mRNA vaccine	mRNA	19	1	Ongoing
	TV	NCT00078494	CC	LMP2:340–349 + LMP2:419–427	Peptide	99	1	Completed
	TV	NCT01094405	Chinese U of Hong Kong	MVA EBNA1/LMP2	Viral Vectored	25	2	Completed
HBV	TV	NCT06513286	Ludwig-Maximilians-University, Munich	TherVacB	Protein-based + Bacterial Vectored	81	1/2	Ongoing
	TV	NCT04297917	Barinthus Biothertapeutics	ChAdOx-1-HBV	Viral Vectored	47	1	Completed
HCV	PV	NCT01436357	NAID	AdCh3NSmut1+ MVA-NSMut	Viral Vectored	548	1/2	Completed
	PV	NCT03688061	U of Oxford	ChAd3-hliNSmut + MVA-hliNSmut	Viral Vectored	25	1	Completed
	PV	NCT01296451	Rei Ther Sri	AdCh3NSmut + MVA-NSmut	Viral Vectored	55	1	Completed
	TV	NCT00601770	Valneva Austria GmbH	IC41	mRNA	71	2	Completed
